# Associations of sleep characteristics with atopic disease: a cross-sectional study among Chinese adolescents

**DOI:** 10.1186/s13223-021-00516-7

**Published:** 2021-02-22

**Authors:** Yiting Chen, Qian Yang, Kena Zhao, Zengqiang Wu, Xiaoming Shen, Shenghui Li

**Affiliations:** 1 School of Public Health, Shanghai Jiao Tong University School of Medicine, 227 South Chongqing Road, Huangpu District, Shanghai, 200025 China; 2 MOE-Shanghai Key Laboratory of Children’s Environmental Health, Shanghai Jiao Tong University School of Medicine, Shanghai, China; 3 Shanghai Academy of Educational Science, Shanghai, China

**Keywords:** Sleep, Asthma, Allergic rhinitis, Eczema, Adolescents

## Abstract

**Background:**

Adolescence, as a transition between childhood and adulthood, is a critical stage for the long-term control of atopic diseases. We aim to determine if sleep characteristics are involved in the increased risk of atopic disease among adolescents.

**Methods:**

Adopting the stratified cluster random sampling method, this cross-sectional survey included 4932 participants aged 12–18 years. The Chinese version of adolescent sleep disturbance questionnaire and the adolescent sleep hygiene scale were used to collect information on sleep problems and sleep hygiene, respectively. Logistic regression models were implemented to examine the associations of sleep with atopic diseases.

**Results:**

Sleep duration was not found to be related with allergic diseases. By contrast, sleep-disordered breathing was associated with an increased risk of asthma (adjusted OR = 1.79, 95% CI 1.25–2.55), allergic rhinitis (adjusted OR = 1.95, 95% CI 1.52–2.49), and eczema (adjusted OR = 1.63, 95% CI 1.23–2.16); poor sleep physiology was correspondent to increased odds of asthma (adjusted OR = 1.69, 95% CI 1.24–2.29), allergic rhinitis (adjusted OR = 1.40, 95% CI 1.13–1.73) and eczema (adjusted OR = 1.66, 95% CI 1.32–2.09); non-optimal sleep environment was associated with an increased prevalence of asthma (adjusted OR = 1.52, 95% CI 1.08–2.12), allergic rhinitis (adjusted OR = 1.32, 95% CI 1.04–1.69) and eczema (adjusted OR = 1.53, 95% CI 1.19–1.96).

**Conclusions:**

As sleep-disordered breathing, poor sleep physiology and non-optimal sleep environment were associated with a higher risk of allergic diseases, the results of this study provide a new concept for the adjuvant treatment of allergic diseases in adolescents. Management strategies of allergic diseases should take regular screening and targeted treatment of sleep issues into account.

## Background

The increasing prevalence of allergic diseases has aroused great public concern in the world [1]. In Shanghai, the prevalence of children’s allergic diseases has increased exponentially in recent decades, predisposing Shanghai to the city of the highest prevalence of allergic diseases in China [2]. It is generally believed that allergic diseases are prevalent mainly in childhood [3]. Studies have shown that allergic diseases usually occur in early childhood, and can persist into adulthood if not controlled promptly [4, 5]. Adolescence is the transitional period between childhood and adulthood. If effective control is carried out at puberty, potential risk factors and a sturdy immune system might work together in a positive way, thus controlling the allergic diseases without recurrence. However, to date, very limited data are available regarding allergic diseases in adolescence [6, 7].

Even though sleep plays an important role in daily functioning, a high prevalence of sleep problems has been reported in adolescence [8, 9]. Accumulating evidence suggested that sleep was implicated in metabolism and immune regulation [10–13]. Recently, new evidence has further suggested that disturbed sleep and atopic disorders are intrinsically linked in pathophysiological processes [14].

A previous cross-sectional study has indicated that sleep duration < 7.8 h per night was associated with the higher odds of food and aeroallergens sensitization in rural Chinese adolescents [15]. Longitudinal studies further suggest that sleep problems, e.g., being overtired, sleep-disordered breathing, and persistent frequent nocturnal waking, were associated with higher risks of allergic rhinitis or asthma in western adolescents, however, it does not result in higher odds of eczema [16]. A prospective observational study conducted in Ohio, USA suggested that sleep-disordered breathing can be a modifiable risk factor for severe asthma, revealing that asthma symptoms could be improved by treating SDB [17]. Although these findings indicated a potential link between sleep and allergic diseases, evidence on adolescence is still scarce. Furthermore, most of the existing studies have assessed only a subset of sleep dimensions, such as sleep duration, sleep problems, or a specific allergic disease. So far as we know, the relationship between all-round sleep features and atopic diseases has not yet been identified. To fill this gap, our research systematically expounded on sleep problems, sleep hygiene, and sleep duration, covering comprehensive aspects of sleep features. Moreover, this study included three major allergic diseases prevalent in adolescents, asthma, allergic rhinitis, and eczema. Finally, sleep patterns on weekdays and weekends were analyzed separately as different school schedules and socioeconomic factors may lead to divergent sleep characteristics among Chinese adolescents and those in Western countries [18].

To the best of our knowledge, this is the first cross-sectional study evaluating the associations of comprehensive sleep characteristics, herein sleep problems, sleep hygiene, and sleep duration, with asthma, allergic rhinitis, and eczema among adolescents.

## Methods

### Study participants and protocol

The present study, based on a cross-sectional survey, was conducted in Shanghai in November 2009. Detailed information on participant recruitment was described previously [19]. Briefly, among 9 urban areas and 8 suburban/rural areas in Shanghai, 4 urban areas and 2 suburb/rural areas were randomly sampled. Subsequently, 2 junior high schools and 2 senior high schools were randomly selected in each area. Overall, 5159 students were eligible for this study. Among them, 96.25% completed the questionnaires. The final sample consisted of 4932 participants after deleting the missing data on targeted allergic diseases.

The survey was conducted in health education classes. The purpose of the survey and guidance on questionnaires were explained to teachers and students, and the principle of voluntary participation was accentuated. Students were asked to finish the questionnaire anonymously, absent students were not counted towards the total.

### Sleep variables

#### Sleep duration

Sleep habit was evaluated by standardized questionnaires. Given the fact that students’ sleep condition on weekends may be differentiated from that on weekdays. Weekday (Monday to Friday) and weekend (Saturday and Sunday) bedtime, wake-up time, and sleep latency time were asked separately, and sleep duration during weekdays and weekends were calculated, respectively [15]. Night-time sleep duration was calculated based on the time a child usually goes to bed at night and wakes up in the morning, which was found to be normally distributed.

#### Sleep problem

The Chinese version of the adolescent sleep disturbance questionnaire (ASDQ) is a 26-item instrument to assess sleep problems of adolescents aged 12–18 years [20]. The ASDQ has been approved to be a qualified instrument for assessing sleep problems among Chinese adolescent in our previous study (Cronbach’s alpha coefficients for the internal consistency were 0.71 for the overall questionnaire and ranged from 0.61 to 0.73 for subscales; intraclass correlation coefficients for the test–retest reliability were 0.85 for the overall questionnaire and ranged from 0.64 to 0.82 for subscales) [21, 22]. The 26 items were conceptually grouped into 6 subscales: difficulty in falling asleep (5 items), difficulty in maintaining sleep (7 items), difficulty in reinitializing sleep (5 items), difficulty in returning to wakefulness (3 items), sleep-disordered breathing (3 items) and disorders of arousal (3 items). The participants reported how often these sleep behaviors in each item occurred in the last month, and a 5-point Likert-type scale (4 = always, 0 = never) was adopted. In each subscale, if any sleep problem occurred once per week or more frequently, we defined that the participant suffered from the corresponding sleep problem. Each subscale score was encoded into two categories: “1” indicating the presence of such sleep problems, and “0” indicating the absence of such sleep problems.

#### Sleep hygiene

The adolescent sleep hygiene scale (ASHS) was adopted to assess sleep hygiene among adolescents [23]. Based on the literature review [24], the ASHS was modified in terms of the current needs. There were 24 items in the modified Chinese version of ASHS (M-ASHS). These are divided into six subgroups by different contents, including sleep physiology, sleep cognition, sleep emotion, sleep environment, and sleep stability, which has been reported in the previous study [22, 25]. 5-point Likert-type scale was implemented (1 = always, 5 = never), that a higher score indicates a higher potential of sleep hygiene situation. M-ASHS for the Chinese version had been validated, showing a strong reliability (for the overall questionnaire, Cronbach’s alpha coefficients for the internal consistency were 0.89 and intraclass correlation coefficients for the test–retest reliability were 0.85. For subscales, internal consistency ranged from 0.88 to 0.91, and intraclass correlation coefficients for the test–retest reliability ranged from 0.60 to 0.88) [22, 25]. In each dimension, if one or more sleep behavior occurred once per week or more frequently, we defined that the participant was stuck in poor sleep hygiene. To evaluate the impact of sleep hygiene on atopic diseases, each subscale score was recoded into two categories: “0” for good sleep hygiene, and “1” for poor sleep hygiene.

### Ascertainment of allergic diseases

Self-reported yes–no questions were applied to evaluate atopic diseases, including asthma, allergic rhinitis, and eczema. The prevalence of asthma, allergic rhinitis, and eczema were based on the participants’ responses to the following three questions: “Have you been diagnosed with asthma/allergic rhinitis/eczema by clinical specialists?”.

### Covariates variables

We also collected participants’ information on possible confounding factors based on the literature [25].

1. Demographic characteristics consist of the age and gender of the participants. Family structures were divided into single-parent families, nuclear families, and extended families; socioeconomic status include the average personal monthly household income and educational levels of parents.
2. Maternal and perinatal characteristics included delivery mode, feeding pattern, mother’s smoking exposure during gestation, mother’s drinking status during gestation, mother’s sleep condition during gestation, mother’s age at delivery, and father’s age at delivery.
3. Biological chronic health problems and activity routine behaviors included overweight/obesity, smoking exposure, drinking status, physical activity, respectively on weekdays and weekends.

### Statistical analysis

The percentage for categorical variables was reported in statistical descriptions. Logistic regression was applied to compare demographic factors between normal and allergic disease groups. Additionally, multivariate logistic regression was implemented to estimate the adjusted associations of sleep problems, sleep hygiene, sleep duration, and three allergic diseases. With “1” for adolescents having a certain kind of allergic disease and “0” for adolescents without any of the three allergic diseases. Adjustments were made following a three-step procedure. First, simply adjusted for demographic characteristics, family structure, and socioeconomic status (Adjusted model 1). Second, based on Adjusted model 1, conditions of gestation, delivery, and feeding were further adjusted (Adjusted model 2). Finally, based on the prior two adjusted models, adolescent’s health problems, daily activity routines were further adjusted (Adjusted model 3). Spearman correlation analysis was used to examine the correlation between sleep parameters and different atopic diseases. The Benjamini and Hochberg false discovery rate method was adopted for multiple testing corrections [26].

All analyses were performed with the Statistical Package for the Social Sciences version 24 (SPSS Inc, Chicago, IL, USA) and the p.adjust package for R statistical software (version 3.5.1). The significance level was set at p < 0.05 for two tails.

## Results

### Descriptive analysis

**Fig. 1:**
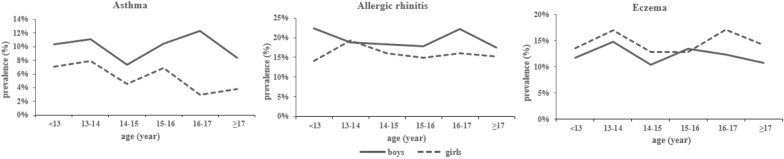
Age and gender-specific prevalence of childhood respiratory allergies

Figure 1 illustrated that the prevalence of allergic diseases was higher in boys than in girls. There were 7.4%, 17.5%, and 13.4% of participants has been diagnosed with asthma, allergic rhinitis, and eczema, respectively. Table 1 summarized the distribution of age, gender, socio-economic status, maternal and perinatal characteristics as well as lifestyles of three patient groups. Generally, children who are male, living in a high-income family, having a mother or/and father of high educational level, been given mixed or/and artificial feeding patterns, and of high maternal and/or paternal age at delivery were associated with an increased prevalence of three allergic diseases. Besides, adolescents who are overweight and get drinks were more likely to develop asthma and allergic rhinitis. Post-term delivery was associated with an increased risk of asthma (*p* = 0.02). Maternal smoking during pregnancy was associated with increased odds of asthma (*p* = 0.05) and allergic rhinitis (*p* = 0.04), and maternal poor sleep during pregnancy was shown to be associated with an increased risk of eczema (*p* = 0.002).

**Table 1:** Characteristics of participants with asthma, allergic rhinitis and eczema, column %

Characteristics (N)	Asthma (N = 367)			Allergic rhinitis (N = 861)			Eczema (N = 661)		
	%	OR (95% CI)		%	OR (95% CI)		%	OR (95% CI)	
Demographic characteristics									
Age (years)									
< 13 (649)	15.3	*0.69 (0.48–0.98)*	*0.04*	13.7	0.88 (0.69–1.13)	0.33	12.4	1.00 (0.75–1.32)	0.98
13–14 (694)	18.0	0.88 (0.59–1.31)	0.53	15.4	1.07 (0.81–1.41)	0.65	16.7	1.19 (0.87–1.63)	0.28
14–15 (707)	11.5	1.00 (0.68–1.47)	0.99	14.1	0.91 (0.68–1.21)	0.45	12.6	1.04 (0.75–1.43)	0.82
15–16 (715)	16.9	0.68 (0.45–1.04)	0.07	13.7	0.94 (0.71–1.25)	0.66	14.3	0.92 (0.66–1.28)	0.61
16–17 (695)	14.2	1.17 (0.80–1.71)	0.41	15.4	1.12 (0.85–1.49)	0.42	15.6	1.34 (0.98–1.83)	0.07
≥17 (1450)	24.0	Ref.		27.7	Ref.		28.4	Ref.	
Gender									
Boy (2422)	64.4	*1.92 (1.53–2.40)*	*< 0.001*	54.0	*1.25 (1.07–1.45)*	*0.004*	44.4	*0.85 (0.72–1.00)*	*0.05*
Girl (2495)	35.6	Ref.		46.0	Ref.		55.6	Ref.	
Household income (RMB^b^/person/month)									
> 3000RMB^b^ (2100)	53	*1.60 (1.28–1.99)*	*< 0.001*	51.3	*1.50 (1.28–1.74)*	*< 0.001*	50.5	*1.45 (1.22–1.71)*	*< 0.001*
≦ 3000RMB^b^ (2694)	47	Ref.		48.7	Ref.		49.5	Ref.	
Family structure									
Single parent family (269)	6.2	1.23 (0.77–1.97)	0.38	6.4	1.22 (0.89–1.69)	0.22	6.0	1.18 (0.82–1.70)	0.37
Nuclear family (2632)	51.7	Ref.		53.8	Ref.		52.2	Ref.	
Extended family (1941)	42.1	1.11 (0.89–1.39)	0.36	39.8	1.01 (0.86–1.18)	0.91	41.8	1.09 (0.92–1.30)	0.33
Mother’s educational levels									
College and above (1713)	47.4	*2.71 (1.97–3.73)*	*< 0.001*	46.3	*2.68 (2.15–3.35)*	*< 0.001*	44.2	*2.32 (1.82–2.95)*	*< 0.001*
High school (1674)	37.0	*2.01 (1.44–2.79)*	*< 0.001*	38.3	*2.11 (1.68–2.64)*	*< 0.001*	38.7	*1.93 (1.51–2.46)*	*< 0.001*
Middle school and below (1,218)	15.6	Ref.		15.4	Ref.		17.0	Ref.	
Father’s educational levels									
College and above (1980)	51.9	*2.90 (2.03–4.15)*	*< 0.001*	52.1	*2.62 (2.07–3.30)*	*< 0.001*	50.6	*2.30 (1.79–2.96)*	*< 0.001*
High school (1731)	36.8	*2.15 (1.49–3.11)*	*< 0.001*	35.3	*1.85 (1.45–2.35)*	*< 0.001*	35.4	*1.68 (1.29–2.18)*	*< 0.001*
Middle school and below (1015)	11.3	Ref.		12.6	Ref.		13.9	Ref.	
Obstetrics and parental health characteristics									
Delivery mode									
Premature delivery (504)	10.6	1.05 (0.73–1.51)	0.79	11.5	1.12 (0.88–1.43)	0.36	12.7	1.25 (0.96–1.62)	0.10
Post-term delivery (297)	9.2	*1.59 (1.07–2.35)*	*0.02*	7.0	1.19 (0.87–1.67)	0.27	6.5	1.12 (0.79–1.59)	0.53
Term delivery (3858)	80.2	Ref.		81.5	Ref.		80.7	Ref.	
Feeding pattern									
Mixed feeding (994)	23.1	*1.34 (1.02–1.76)*	*0.04*	25.7	*1.51 (1.26–1.82)*	*< 0.001*	26.7	*1.58 (1.29–1.94)*	*< 0.001*
Artificial feeding (681)	19.1	*1.59 (1.18–2.14)*	*0.002*	17.4	*1.48 (1.19–1.83)*	*< 0.001*	17.0	*1.45 (1.14–1.84)*	*0.002*
Breastfeeding (2991)	57.8	Ref.		56.8	Ref.		56.3	Ref.	
Maternal smoking during gestation									
Yes (40)	1.7	*2.48 (1.00–6.13)*	*0.05*	1.4	*2.12 (1.05–4.27)*	*0.04*	0.8	1.16 (0.44–3.05)	0.77
No (4756)	98.3	Ref.		98.6	Ref.		99.2	Ref.	
Maternal drinking during gestation									
Drinking (71)	1.7	1.18 (0.50–2.78)	0.70	1.9	1.35 (0.76–2.39)	0.30	1.3	0.88 (0.42–1.88)	0.75
Non-drinking (4721)	98.3	Ref.		98.1	Ref.		98.8	Ref.	
Mother’s sleep condition during gestation									
Fair (1354)	27.2	0.98 (0.78–1.23)	0.85	30.3	1.12 (0.96–1.30)	0.16	34.8	*1.31 (1.11–1.55)*	*0.002*
Good (3227)	72.8	Ref.		69.7	Ref.		65.2	Ref.	
Mother’s age at delivery(year)									
≥ 30 (644)	19.5	*1.99 (1.45–2.73)*	*< 0.001*	17.2	*1.60 (1.27–2.00)*	*< 0.001*	17.2	*1.58 (1.23–2.04)*	*< 0.001*
25–29 (1870)	47.5	*1.64 (1.28–2.10)*	*< 0.001*	45.5	*1.43 (1.21–1.69)*	*< 0.001*	45.9	*1.43 (1.18–1.73)*	*< 0.001*
≤ 24 (2057)	33.0	Ref.		37.3	Ref.		36.9	Ref.	
Father’s age at delivery									
≥ 35 (486)	14.4	*2.16 (1.56–2.98)*	*< 0.001*	11.6	*1.69 (1.34–2.12)*	*< 0.001*	13.3	*1.71 (1.32–2.21)*	*< 0.001*
25–34 (2971)	76.4	*1.79 (1.39–2.30)*	*< 0.001*	74.3	*1.52 (1.28–1.81)*	*< 0.001*	69.7	*1.55 (1.28–1.87)*	*< 0.001*
≤ 24 (1037)	9.2	Ref.		14.1	Ref.		13.2	Ref.	
Biological chronic health problems and activity routine behaviors									
Overweight/obesity									
Yes (659)	19.4	*1.59 (1.20–2.11)*	*0.001*	17.1	*1.36 (1.10–1.67)*	*0.004*	15.5	1.21 (0.95–1.54)	0.12
No (4009)	80.6	Ref.		82.9	Ref.		84.5	Ref.	
Smoking exposure									
Yes (1313)	30.5	1.23 (0.97–1.56)	0.08	29.3	1.17 (0.99–1.38)	0.07	29.3	1.16 (0.97–1.40)	0.11
No (3522)	69.5	Ref.		70.7	Ref.		70.7	Ref.	
Drinking status									
Current drinking (381)	11.7	*1.73 (1.22–2.43)*	*0.002*	10.0	*1.45 (1.12–1.87)*	*0.005*	8.3	1.18 (0.87–1.60)	0.29
Non-drinking (4540)	88.3	Ref.		90.0	Ref.		91.7	Ref.	
Physical activity during weekdays									
Physically active time < 1 h/day (4049)	68.7	0.95 (0.71–1.26)	0.71	67.8	1.02 (0.84–1.25)	0.84	70.3	0.95 (0.76–1.18)	0.62
Physically active time ≥ 1 h/day (843)	31.3	Ref.		32.2	Ref.		29.7	Ref.	
Physical activity during weekends									
Physically active time < 1 h/day (3421)	81.9	0.94 (0.74–1.19)	0.60	83.0	0.90 (0.77–1.06)	0.20	81.9	1.01 (0.84–1.22)	0.90
Physically active time ≥ 1 h/day (1470)	18.1	Ref.		17.0	Ref.		18.1	Ref.	

^**a**^Statistically significant results (p < 0.05) are in italics

^b^RMB, China’s currency (yuan)

### Association between sleep features and allergic diseases

Table 2 summarized all sleep characteristics and their crude relationships with three allergic diseases. For asthma, sleep-disordered breathing and sleep physiology were found to be influential factors after adjusting for multiple testing. Difficulty in maintaining sleep (*p* = 0.002, p* = 0.008), sleep-disordered breathing (*p* < 0.001, p* < 0.001), poor sleep physiology (*p* = 0.001, p* = 0.008) and sleep emotion (*p* = 0.002, p* = 0.008) were all associated with an increased risk of allergic rhinitis, even after adjusting for multiple comparisons. Except for difficulty in falling asleep (*p* = 0.09, p* = 0.18), all other sleep parameters on sleep problems and sleep hygiene were found to be associated with higher risks of eczema in the univariate regression models. When it comes to sleep duration, only sleep < 8 h on weekend was associated with an increased risk of eczema (*p* = 0.03, p* = 0.04).

**Table 2:** Sleep characteristics of participants with asthma, allergic rhinitis and eczema, column %

Characteristics (N)	Asthma				Allergic rhinitis				Eczema			
	%	OR (95% CI)	*p*	p*	%	OR (95% CI)	*p*	p*	%	OR (95% CI)	*p*	p*
Sleep problem												
Difficulty in falling asleep												
Yes (2910)	55.9	0.92 (0.71–1.20)	0.55	0.84	56.6	0.85 (0.71–1.01)	0.07	0.12	63.4	1.19 (0.98–1.46)	0.09	0.18
No (2022)	44.1	Ref.			43.4	Ref.			36.6	Ref.		
Difficulty in maintaining sleep												
Yes (3342)	69.8	1.23 (0.93–1.63)	0.14	0.35	73.4	*1.36 (1.12–1.65)*	*0.002*	*0.008*	75.8	*1.73 (1.38–2.17)*	*< 0.001*	*< 0.001*
No (1590)	30.2	Ref.			26.6	Ref.			24.2	Ref.		
Difficulty in reinitializing sleep												
Yes (2117)	44.7	1.08 (0.83–1.40)	0.56	0.84	45.6	1.19 (1.00–1.42)	0.06	0.12	51.9	*1.62 (1.33–1.97)*	*< 0.001*	*< 0.001*
No (2815)	55.3	Ref.			54.4	Ref.			48.1	Ref.		
Difficulty in returning to wakefulness												
Yes (4341)	86.1	0.89 (0.62–1.28)	0.54	0.84	80.4	1.20 (0.92–1.57)	0.19	0.26	90.0	*1.44 (1.04–1.98)*	*0.03*	*0.04*
No (591)	13.9	Ref.			11.6	Ref.			10.0	Ref.		
Sleep-disordered breathing												
Yes (694)	22.6	*1.93 (1.38–2.70)*	*< 0.001*	*< 0.001*	20.4	*1.96 (1.55–2.48)*	* < 0.001*	* < 0.001*	17.4	*1.67 (1.24–2.13)*	*< 0.001*	*< 0.001*
No (4238)	77.4	Ref.			79.6	Ref.			82.6	Ref.		
Disorders of arousal												
Yes (1271)	25.9	1.07 (0.79–1.44)	0.67	0.92	28.3	1.21 (0.99–1.48)	0.06	0.12	33.7	*1.60 (1.30–1.98)*	*< 0.001*	*< 0.001*
No (3661)	74.1	Ref.			71.7	Ref.			66.3	Ref.		
Sleep hygiene												
Sleep physiology												
Bad (1171)	28.9	*1.65 (1.24–2.20)*	*0.001*	*0.008*	28.2	*1.40 (1.14–1.72)*	*0.001*	*0.008*	31.5	*1.69 (1.36–2.10)*	*< 0.001*	*< 0.001*
Good (3701)	71.1	Ref.			71.8	Ref.			68.5	Ref.		
Sleep cognition												
Bad (994)	24.4	*1.43 (1.05–1.95)*	*0.02*	0.11	22.5	1.20 (0.96–1.50)	0.11	0.16	28.1	*1.81 (1.44–2.27)*	*< 0.001*	*< 0.001*
Good (3822)	75.6	Ref.			77.5	Ref.			71.9	Ref.		
Sleep emotion												
Bad (1018)	23.8	*1.23 (0.89–1.69)*	0.21	0.45	24.5	*1.40 (1.13–1.73)*	*0.002*	*0.008*	27.9	*1.79 (1.43–2.24)*	* < 0.001*	* < 0.001*
Good (3890)	76.2	Ref.			75.5	Ref.			72.1	Ref.		
Sleep environment												
Bad (914)	21.1	*1.39 (1.01–1.91)*	*0.04*	0.13	20.5	*1.26 (1.00–1.57)*	*0.05*	0.12	24.8	*1.48 (1.16–1.88)*	*0.001*	*0.002*
Good (3993)	78.9	Ref.			79.5	Ref.			75.2	Ref.		
Sleep stability												
Bad (1008)	25.1	*1.41 (1.04–1.91)*	*0.03*	0.11	22.3	1.21 (0.98–1.51)	0.08	0.14	24.8	*1.46 (1.15–1.84)*	*0.002*	*0.003*
Good (3796)	74.9	Ref.			77.7	Ref.			75.2	Ref.		
Sleep duration												
Nighttime sleep duration in weekday												
< 8 h/day (2466)	50.3	0.96 (0.57–1.61)	0.87	0.94	50.8	0.96 (0.67–1.37)	0.82	0.88	54.2	1.40 (0.91–2.16)	0.13	0.14
8–9 h/day (2060)	43.1	0.96 (0.57–1.63)	0.89	0.94	42.5	0.92 (0.65–1.32)	0.66	0.77	40.4	1.12 (0.73–1.74)	0.60	0.64
> 9 h/day (307)	6.5	Ref.			6.7	Ref.			5,4	Ref.		
Nighttime sleep duration on weekend												
< 8 h/day (468)	9.4	1.05 (0.66–1.67)	0.84	0.94	9.6	1.00 (0.72–1.38)	1.00	1.00	11.3	*1.44 (1.04–1.99)*	*0.03*	*0.04*
8–9 h/day (1970)	41.0	0.99 (0.75–1.30)	0.94	0.94	40.7	0.94 (0.78–1.13)	0.49	0.61	40.5	1.03 (0.83–1.27)	0.79	0.79
> 9 h/day (2369)	49.6	Ref.			49.6	Ref.			48.1	Ref.		

^a^Statistically significant results (p < 0.05) are in italics

^*^p value corrected

As shown in Table 3, sleep duration on weekdays and weekends was not associated with risks of asthma and allergic rhinitis. Nighttime sleep < 8 h on weekends was associated with higher odds of eczema in the final adjusted model (adjusted OR: 1.40, 95% CI 1.00–1.97, *p* = 0.05), however, the significance disappeared after correcting for multiple comparisons (*p* = 0.07). Table 4 showed associations between six subscales of sleep problems with allergic diseases. Difficulty in falling asleep (adjusted OR: 1.26, 95% CI 1.03–1.55, *p* < 0.001) and disorders of arousal (adjusted OR: 1.55, 95% CI 1.24–1.92, *p* < 0.001) were associated with an increased risk of eczema. Difficulties in maintaining sleep and reinitializing sleep were also significantly associated with allergic rhinitis and eczema in the final model. Meanwhile, sleep-disordered breathing was associated with 79% (95% CI 1.25–2.55, *p* = 0.001), 95% (95% CI 1.52–2.49, *p* < 0.001) and 63% (95% CI 1.23–2.16, *p* = 0.001) higher odds of asthma, allergic rhinitis, and eczema, respectively. Table 5 showed that poor sleep psychology and sleep environment were associated with a predisposition to developing all three allergic diseases in final adjusted models. Negative sleep cognition and poor sleep stability presented a significant association with higher odds of asthma and eczema. Negative sleep emotion was significantly associated with higher risks of allergic rhinitis (adjusted OR = 1.36, 95% CI 1.09–1.70, *p* = 0.006) and eczema (adjusted OR = 1.71, 95% CI 1.36–2.16, *p* < 0.001). Since significant correlations were identified between sleep parameters as well as between atopic diseases (Additional file 1: Table S1, S2), multiple testing corrections were performed. And most of the significance remained after correcting for multiple comparisons.

**Table 3:** Association of sleep duration with asthma, allergic rhinitis, eczema

	Sleep duration, odds ratio (95% confidence interval)											
	Nighttime sleep duration in weekday						Nighttime sleep duration on weekends					
	< 8 vs. ≥ 9 h/day	*p*	p*	8–9 vs. ≥ 9 h/day	*p*	p*	< 8 vs. ≥ 9 h/day	*p*	p*	8–9 vs. ≥ 9 h/day	*p*	p*
Asthma												
Adjusted model1	1.19 (0.67–2.12)	0.55	0.83	0.99 (0.58–1.70)	0.98	0.98	1.01 (0.63–1.64)	0.96	0.98	1.02 (0.77–1.35)	0.91	0.98
Adjusted model 2	1.22 (0.68–2.17)	0.51	0.77	1.03 (0.60–1.78)	0.91	0.96	1.03 (0.64–1.68)	0.90	0.96	1.04 (0.78–1.38)	0.81	0.96
Adjusted model 3	1.19 (0.66–2.13)	0.57	1.00	1.05 (0.61–1.80)	0.88	1.00	0.97 (0.60–1.59)	0.91	1.00	1.04 (0.78–1.38)	0.80	1.00
Allergic rhinitis												
Adjusted model1	0.92 (0.62–1.37)	0.69	0.74	0.87 (0.60–1.26)	0.46	0.53	0.95 (0.69–1.33)	0.78	0.78	0.93 (0.76–1.12)	0.44	0.53
Adjusted model 2	0.92 (0.62–1.37)	0.69	0.74	0.88 (0.61–1.28)	0.51	0.58	0.96 (0.68–1.34)	0.79	0.79	0.93 (0.76–1.13)	0.46	0.57
Adjusted model 3	0.91 (0.61–1.36)	0.64	0.69	0.88 (0.61–1.28)	0.50	0.58	0.94 (0.67–1.31)	0.70	0.70	0.93 (0.76–1.13)	0.45	0.56
Eczema												
Adjusted model1	1.49 (0.93–2.40)	0.10	0.11	1.17 (0.74–1.83)	0.50	0.54	1.40 (1.00–1.95)	0.05	0.07	1.03 (0.83–1.28)	0.70	0.77
Adjusted model 2	1.45 (0.90–2.34)	0.12	0.14	1.16 (0.74–1.82)	0.53	0.56	*1.44 (1.02–2.02)*	*0.04*	*0.05*	1.05 (0.85–1.31)	0.65	0.65
Adjusted model 3	1.45 (0.90–2.34)	0.13	0.15	1.16 (0.74–1.82)	0.52	0.56	*1.40 (1.00–1.97)*	*0.05*	0.07	1.05 (0.85–1.31)	0.65	0.65

Adjusted model 1 was adjusted for demographic characteristics, family structure and socioeconomic status

Adjusted model 2 was further adjusted for conditions of gestation, delivery and feeding besides the covariates in adjusted model 1

Adjusted model 3 was further adjusted for health problems, daily activity and behavior routine besides the covariates in adjusted model2

^*^p value corrected

^**a**^Statistically significant results (*p* < 0.05) are in italics

**Table 4:** Association of sleep problems with asthma, allergic rhinitis, eczema

	Sleep problems, odds ratio (95% confidence interval)																	
	Subscale 1	*p*	p*	Subscale 2	*p*	p*	Subscale 3	*p*	p*	Subscale 4	*p*	p^*^	Subscale 5	*p*	p*	Subscale 6	*p*	p*
Asthma																		
Adjusted model 1	0.98 (0.75–1.27)	0.85	0.98	1.26 (0.95–1.67)	0.11	0.27	1.16 (0.89–1.51)	0.28	0.52	1.01 (0.69–1.46)	0.98	0.98	*1.90 (1.34–2.69)*	*< 0.001*	*< 0.001*	1.12 (0.83–1.52)	0.46	0.77
Adjusted model 2	1.01 (0.77–1.32)	0.96	0.96	1.28 (0.96–1.70)	0.09	0.23	1.17 (0.90–1.53)	0.25	0.47	0.99 (0.68–1.44)	0.95	0.96	*1.87 (1.32–2.66)*	*< 0.001*	*< 0.001*	1.11 (0.82–1.51)	0.51	0.77
Adjusted model 3	1.00 (0.77–1.31)	0.98	1.00	1.25 (0.94–1.67)	0.12	0.30	1.14 (0.87–1.50)	0.34	0.63	0.96 (0.65–1.40)	0.82	1.00	*1.79 (1.25–2.55)*	*0.001*	*0.008*	1.08 (0.79–1.47)	0.65	1.00
Allergic rhinitis																		
Adjusted model 1	0.87 (0.72–1.04)	0.12	0.18	*1.38 (1.14–1.68)*	*0.001*	*0.005*	*1.25 (1.05–1.50)*	*0.02*	*0.04*	1.24(0.93–1.63)	0.14	0.19	*2.01 (1.57–2.56)*	*< 0.001*	*< 0.001*	*1.24 (1.01–1.52)*	*0.04*	0.08
Adjusted model 2	0.87 (0.72–1.04)	0.12	0.19	*1.38 (1.13–1.68)*	*0.001*	*0.005*	*1.25 (1.04–1.50)*	*0.02*	*0.04*	1.23 (0.93–1.63)	0.15	0.21	*1.99 (1.56–2.55)*	*< 0.001*	*< 0.001*	*1.23 (1.00–1.51)*	*0.05*	0.10
Adjusted model 3	0.86 (0.72–1.04)	0.11	0.19	*1.36 (1.12–1.66)*	*0.002*	*0.01*	*1.24 (1.04–1.49)*	*0.02*	*0.05*	1.22 (0.92–1.61)	0.18	0.25	*1.95 (1.52–2.49)*	*< 0.001*	*< 0.001*	1.21 (0.99–1.49)	0.07	0.14
Eczema																		
Adjusted model 1	*1.25 (1.02–1.53)*	*0.04*	*0.05*	*1.78 (1.42–2.23)*	*< 0.001*	*< 0.001*	*1.70 (1.39–2.08)*	*< 0.001*	*< 0.001*	*1.39 (1.00–1.94)*	*0.05*	0.06	*1.69 (1.28–2.24)*	*< 0.001*	*< 0.001*	*1.58 (1.27–1.96)*	*< 0.001*	*< 0.001*
Adjusted model 2	*1.26 (1.02–1.55)*	*0.03*	*0.04*	*1.77 (1.41–2.23)*	*< 0.001*	*< 0.001*	*1.72 (1.40–2.10)*	*< 0.001*	*< 0.001*	*1.40 (1.00–1.95)*	*0.05*	0.06	*1.69 (1.28–2.23)*	*< 0.001*	*< 0.001*	*1.57 (1.26–1.94)*	*< 0.001*	*< 0.001*
Adjusted model 3	*1.26 (1.03–1.55)*	*0.03*	*0.04*	*1.76 (1.40–2.22)*	*< 0.001*	*< 0.001*	*1.70 (1.39–2.08)*	*< 0.001*	*< 0.001*	1.39(0.99–1.93)	0.06	0.07	*1.63 (1.23–2.16)*	*0.001*	*0.002*	*1.55 (1.24–1.92)*	*< 0.001*	*< 0.001*

Adjusted model 1 was adjusted for demographic characteristics, family structure and socioeconomic status

Adjusted model 2 was further adjusted for conditions of gestation, delivery and feeding besides the covariates in adjusted model 1

Adjusted model 3 was further adjusted for health problems, daily activity and behavior routine besides the covariates in adjusted model2

Subscale 1, difficulty in falling asleep; Subscale 2, difficulty in maintaining sleep; Subscale 3, difficulty in reinitializing sleep; Subscale 4, difficulty in returning to wakefulness; Subscale 5, sleep-disordered breathing; Subscale 6, disorders of arousal

^*^p value corrected

^**a**^Statistically significant results (p < 0.05) are in italics

**Table 5:** Association of sleep hygiene with asthma, allergic rhinitis, eczema

	Sleep hygiene, odds ratio (95% confidence interval)														
	Subscale 1	*p*	p*	Subscale 2	*p*	p*	Subscale 3	*p*	p*	Subscale 4	*p*	p*	Subscale 5	*p*	p*
Asthma															
Adjusted model 1	*1.73 (1.29–2.33)*	*< 0.001*	*< 0.001*	*1.55 (1.12–2.13)*	*0.008*	*0.02*	1.27 (0.92–1.76)	0.15	0.32	*1.56 (1.12–2.17)*	*0.008*	*0.02*	*1.56 (1.14–2.15)*	*0.006*	*0.02*
Adjusted model 2	*1.75 (1.30–2.37)*	*< 0.001*	*< 0.001*	*1.54 (1.12–2.13)*	*0.009*	*0.03*	1.24 (0.90–1.73)	0.19	0.42	*1.56 (1.12–2.18)*	*0.008*	*0.03*	*1.57 (1.13–2.16)*	*0.006*	*0.03*
Adjusted model 3	*1.69 (1.24–2.29)*	*< 0.001*	*< 0.001*	*1.48 (1.07–2.07)*	*0.02*	0.06	1.22 (0.88–1.70)	0.24	0.51	*1.52 (1.08–2.12)*	*0.02*	0.06	*1.50 (1.08–2.08)*	*0.02*	0.06
Allergic rhinitis															
Adjusted model 1	*1.41 (1.14–1.74)*	*0.001*	*0.005*	1.23 (0.97–1.54)	0.08	0.13	*1.41 (1.14–1.76)*	*0.002*	*0.008*	*1.35 (1.07–1.70)*	*0.01*	*0.03*	1.24 (0.99–1.55)	0.06	0.12
Adjusted model 2	*1.41 (1.14–1.75)*	*0.001*	*0.005*	1.20 (0.95–1.51)	0.12	0.19	*1.38 (1.11–1.72)*	*0.004*	*0.02*	*1.34 (1.06–1.69)*	*0.02*	*0.04*	1.23 (0.98–1.55)	0.07	0.13
Adjusted model 3	*1.40 (1.13–1.73)*	*0.002*	*0.01*	1.17 (0.93–1.48)	0.19	0.25	*1.36 (1.09–1.70)*	*0.006*	*0.02*	*1.32 (1.04–1.69)*	*0.02*	*0.005*	1.23 (0.98–1.54)	0.08	0.15
Eczema															
Adjusted model 1	*1.67 (1.33–2.08)*	*< 0.001*	*< 0.001*	*1.82 (1.44–2.30)*	*< 0.001*	* < 0.001*	*1.77 (1.41–2.22)*	*< 0.001*	*< 0.001*	*1.55 (1.21–1.98)*	*< 0.001*	*< 0.001*	*1.42 (1.12–1.80)*	*0.004*	*0.007*
Adjusted model 2	*1.68 (1.34–2.10)*	*< 0.001*	*< 0.001*	*1.82 (1.44–2.29)*	*< 0.001*	* < 0.001*	*1.73 (1.37–2.17)*	*0.001*	*< 0.001*	*1.54 (1.20–1.97)*	*0.001*	*0.002*	*1.43 (1.12–1.82)*	*0.004*	*0.007*
Adjusted model 3	*1.66 (1.32–2.09)*	*< 0.001*	*< 0.001*	*1.78 (1.41–2.26)*	*< 0.001*	* < 0.001*	*1.71 (1.36–2.16)*	*< 0.001*	*< 0.001*	*1.53 (1.19–1.96)*	*0.001*	*0.002*	*1.41 (1.11–1.81)*	*0.005*	*0.008*

Adjusted model 1 was adjusted for demographic characteristics, family structure and socioeconomic status

Adjusted model 2 was further adjusted for conditions of gestation, delivery and feeding besides the covariates in adjusted model 1

Adjusted model 3 was further adjusted for health problems, daily activity and behavior routine besides the covariates in adjusted model2

Subscale 1, sleep physiology; Subscale 2, sleep cognition; Subscale 3, sleep emotion; Subscale 4, sleep environment; Subscale 5, sleep stability

^*^p value corrected

^**a**^Statistically significant results (*p* < 0.05) are in italics

## Discussion

The study assessed the association of sleep duration, sleep problems, and sleep hygiene with three allergic diseases, including asthma, allergic rhinitis, and eczema among adolescents. The study covered a full-spectrum of sleep features, and sleep duration was classified into weekends and weekdays. Variables on sleep problems and sleep hygiene were collected through standardized and validated questionnaires. We found that sleep problems and sleep hygiene, especially sleep-disordered breathing, sleep physiology, and sleep environment, were essentially connected with all atopic diseases in adolescents. These findings provided a comprehensive insight into the relationship between sleep and allergic diseases. Given that adolescence is a critical period for long-term control of atopic episodes, particular emphasis should be placed on sleep problems and sleep hygiene in the management of allergic diseases.

### Sleep duration with atopic adolescents

In our study, short sleep duration was not associated with childhood allergic diseases, which was not exactly consistent with some of the previous findings [27–29]. In a Korean study aiming at adolescents aged 12–18, the odds ratio of allergic rhinitis increased in both genders when sleep duration was < 7 h [27]. A case–control study conducted in Australia, with an enrollment of children aged 5–17 years old, found that male asthmatic children have a significantly shorter sleep duration than controls (425.9 ± 5.4 min vs 441.85 ± 5.4 min), but the difference was not revealed in females [28]. However, a study carried out in the USA obtained no significant difference in total sleep duration between asthmatic and non-asthmatic adolescents on weekdays and weekends [29]. Analogously, we also analyzed weekday and weekend sleep duration separately. Since Chinese high school students were always with lumbersome academic burden, the sleep pattern could vary from weekdays to weekends, as such, it should not be lumped together. This could explain that our results were, in some aspects, different from those in other countries.

### Sleep problems with atopic adolescents

All six subscales of sleep problems were associated with increased odds of eczema among adolescents; and meanwhile, difficulty in maintaining sleep and reinitializing sleep presented to be related to significant higher odds of allergic rhinitis. Our study found that sleep-disordered breathing was associated with increased risks of all three allergic diseases, which was accordant with 12 out of 18 articles’ results on allergic rhinitis reviewed in 2013 [30]. A birth cohort study of 566 toddlers, also indicated that sleep disorder was a potential risk factor for allergic diseases [31]. A case–control study found that compared to controls, participants (6–16-year-old) with eczema had more disturbed sleep with significantly higher scores on initiating and maintaining sleep disorders, excessive daytime sleepiness, and total sleep disturbance [32]. Within same population, children with eczema had worse sleep quality [33]. Similarly, another research in the US suggested that children with eczema had nearly 50% higher odds of reporting sleep-quality disturbances [34]. However, two other cohort studies obtained significant results, not in eczema, but in asthma and allergic rhinitis [16, 17].

### Sleep hygiene with atopic adolescents

For sleep hygiene, adolescents with allergic diseases have more negative bedtime cognitions, feeling worried and uncomfortable while attempting to fall asleep. A review illustrated that children with atopic disease had more nocturnal symptoms, and they might have poorer sleep hygiene for fear of the nocturnal episodes and can be more likely to experience physical arousal [25]. A study which has investigated 298 adolescents found that adolescents with severe asthma were more likely to sleep on someone else’s bed, such as a parent’s bed, sibling’s bed, and even couch, and had more negative bedtime cognition that may interfere with sleep onset [29]. A study involving 549 college students suggested that, after the 8 weeks of education on sleep hygiene, participants tended to maintain more regular sleep schedules, woke up earlier during the week, took fewer naps, and stopped using electronic devices at bedtime [35]. This suggested that sleep hygiene education can benefit adolescents with allergic diseases feasibly and inexpensively.

### Bidirectional mechanisms between sleep and allergic diseases

The causal relationship between sleep and allergic diseases can vary by study, that is, the chicken-and-egg situation. Dating back to 1992, the first study reported the relationship between habitual snoring and exercise-induced asthma [36]. Since then, several studies have attempted to explain the potential mechanism between sleep and allergic diseases [37–42].

Since cortisol secretion, cytokine production, and immune function were all supposed to be regulated by the circadian rhythm [37]. Night-induced anti-inflammatory cytokines such as IL-4 and IL-10 were reported to inhibit sleep, while pro-inflammatory cytokines such as IL-1β, IL-2, TNF, IFN-γ and IL-6 were suggested to promote sleep [38]. On the contrary, sleep also has an impact on the immune function. Sleep loss altered the immune response to lipopolysaccharide stimulations and susceptibility to infections [39–41]. In an animal model, sleep deprivation resulted in increased levels of pro-inflammatory cytokines such as IL-1β, IL-6, and IL-12, and a decreased levels of anti-inflammatory cytokines such as IL-10, which normalized after a 48-h sleep rebound [42]. Studies have shown that the level of IL-1β, IL-6, IL-17, and high sensitivity C-reactive protein increased after chronic partial sleep deprivation, and IL-1β, IL-6, TNF-α levels increased after acute sleep deprivation [40]. Through the variation of cytokine levels, the relationship between sleep disorders and allergic diseases can be bidirectional.

### Limitations

A number of limitations should be noticed. Firstly, our cross-sectional study was incapable of explaining the causal links. The relationship between sleep and allergic disease could be bidirectional, and both are worth further exploration. Additionally, although our study included a large number of confounding factors of the three allergic diseases, information on drug usages were unavailable [43], which should be taken into consideration in future researches. Moreover, the results we present were according to self-reported data, however, atopic diseases were determined by the physician’s diagnosis, and sleep characteristics were based on validated questionnaires. Finally, the results were backed up by data from 10 years ago. Although there has been growing evidence recently, indicating that sleep can play a role in immune regulation, population-based study is largely lacking. Given that our study is aimed to explore the relationship between sleep characteristics and the risk of atopic diseases, rather than describing sleep characteristics and prevalence of the atopic disease, the findings could still provide some implications for further investigations. Future researches can build on our study with more biophysical and biochemical parameters of atopic diseases and more objective measurements of sleep.

## Conclusions

This study is the first to explore the relationship between the full-spectrum sleep features of adolescents, including sleep duration, sleep problem and sleep hygiene, and the three core allergic diseases herein asthma, allergic rhinitis, and eczema. Generally, sleep-disordered breathing, poor sleep physiology, and non-optimal environment were associated with increased risks of all three allergic diseases. This finding provides a new perspective for that sleep may be a modifiable risk factor in the management of the allergic diseases. Regular screening and specific treatment for sleep issues are supposed to be taken into the prevention strategies of allergic diseases.

## Supplementary Information

**Additional file 1: Table S1**. Correlation analysis of sleep parameters. **Table S2** Correlation analysis of allergic diseases.

## Data Availability

The datasets used and analysed during the current study are available from the corresponding author on reasonable request.

## Abbreviations

OR: Odds ratio
CI: Confidence interval
ASDQ: Adolescent sleep disturbance questionnaire
ASHS: Adolescent sleep hygiene scale
M-ASHS: Modified Chinese version of adolescent sleep hygiene scale
IL: Interleukin
TNF: Tumor necrosis factor
IFN-γ: Interferon-γ
