# Supplementary material for: Associations of sleep characteristics with atopic disease: a cross-sectional study among Chinese adolescents
Source: Allergy Asthma Clin Immunol. 2021 Feb 22;17:21. doi: 10.1186/s13223-021-00516-7 (PMC7898413; doi:10.1186/s13223-021-00516-7)
Supplement: Supplementary file 1 — Additional file 1: Table S1. Correlation analysis of sleep parameters. Table S2 Correlation analysis of allergic diseases. [file 13223_2021_516_MOESM1_ESM.docx]

| **Table S1** Correlation analysis of sleep parameters | | | | | | | | | | | | | |
| --- | --- | --- | --- | --- | --- | --- | --- | --- | --- | --- | --- | --- | --- |
|  | **Sleep problem** | | | | | | **Sleep hygiene** | | | | | **Sleep duration** | |
| **Sleep problem** | Subscale 1 | Subscale 2 | Subscale 3 | Subscale 4 | Subscale 5 | Subscale 6 | Subscale 1 | Subscale 2 | Subscale 3 | Subscale 4 | Subscale 5 | Subscale 1 | Subscale 2 |
| Subscale 1 | 1.000 | **.176** | **.218** | **.054** | **.059** | **.094** | **.124** | **.219** | **.183** | **.174** | **.094** | **-.036** | **-.041** |
| *p*-value |  | **<.001** | **<.001** | **<.001** | **<.001** | **<.001** | **<.001** | **<.001** | **<.001** | **<.001** | **<.001** | **.013** | **.004** |
| Subscale 2 |  | 1.000 | **.168** | **.129** | **.149** | **.187** | **.179** | **.207** | **.210** | **.147** | **.106** | **-.043** | .003 |
| *p*-value |  |  | **<.001** | **<.001** | **<.001** | **<.001** | **<.001** | **<.001** | **<.001** | **<.001** | **<.001** | **.003** | .828 |
| Subscale 3 |  |  | 1.000 | .026 | **.123** | **.255** | **.206** | **.252** | **.274** | **.204** | **.108** | **-.032** | **-.060** |
| *p*-value |  |  |  | .067 | **<.001** | **<.001** | **<.001** | **<.001** | **<.001** | **<.001** | **<.001** | **.025** | **<.001** |
| Subscale 4 |  |  |  | 1.000 | **.076** | **.079** | **.128** | **.117** | **.118** | **.085** | **.163** | **-.174** | -.013 |
| *p*-value |  |  |  |  | **<.001** | **<.001** | **<.001** | **<.001** | **<.001** | **<.001** | **<.001** | **<.001** | .385 |
| Subscale 5 |  |  |  |  | 1.000 | **.211** | **.153** | **.139** | **.184** | **.137** | **.072** | **-.071** | **-.037** |
| *p*-value |  |  |  |  |  | **<.001** | **<.001** | **<.001** | **<.001** | **<.001** | **<.001** | **<.001** | **.010** |
| Subscale 6 |  |  |  |  |  | 1.000 | **.218** | **.200** | **.266** | **.188** | **.142** | **-.049** | -.005 |
| *p*-value |  |  |  |  |  |  | **<.001** | **<.001** | **<.001** | **<.001** | **<.001** | **.001** | .753 |
| **Sleep hygiene** |  |  |  |  |  |  |  |  |  |  |  |  |  |
| Subscale 1 |  |  |  |  |  |  | 1.000 | **.384** | **.309** | **.288** | **.246** | **-.138** | **-.049** |
| *p*-value |  |  |  |  |  |  |  | **<.001** | .**<.001** | **<.001** | **<.001** | **<.001** | **.001** |
| Subscale 2 |  |  |  |  |  |  |  | 1.000 | **.366** | **.372** | **.264** | **-.118** | **-.073** |
| *p*-value |  |  |  |  |  |  |  |  | **<.001** | **<.001** | **<.001** | **<.001** | **<.001** |
| Subscale 3 |  |  |  |  |  |  |  |  | 1.000 | **.298** | **.157** | **-.105** | **-.064** |
| *p*-value |  |  |  |  |  |  |  |  |  | **<.001** | **<.001** | **<.001** | **<.001** |
| Subscale 4 |  |  |  |  |  |  |  |  |  | 1.000 | **.177** | **-.060** | -.025 |
| *p*-value |  |  |  |  |  |  |  |  |  |  | **<.001** | **<.001** | .087 |
| Subscale 5 |  |  |  |  |  |  |  |  |  |  | 1.000 | **-.136** | .021 |
| *p*-value |  |  |  |  |  |  |  |  |  |  |  | **<.001** | .145 |
| **Sleep duration** |  |  |  |  |  |  |  |  |  |  |  |  |  |
| Subscale 1 |  |  |  |  |  |  |  |  |  |  |  | 1.000 | **.279** |
| *p*-value |  |  |  |  |  |  |  |  |  |  |  |  | **<.001** |
| Subscale2 |  |  |  |  |  |  |  |  |  |  |  |  | 1.000 |

**Sleep problem:** Subscale 1, difficulty in falling asleep; Subscale 2, difficulty in maintaining sleep; Subscale 3, difficulty in reinitializing sleep; Subscale 4, difficulty in returning to wakefulness; Subscale 5, sleep-disordered breathing; Subscale 6, disorders of arousal;

**Sleep hygiene:** Subscale 1, sleep physiology; Subscale 2, sleep cognition; Subscale 3, sleep emotion; Subscale 4, sleep environment; Subscale 5, sleep stability;

**Sleep duration:** Subscale 1: nighttime sleep duration in weekday; Subscale 2: nighttime sleep duration on weekend.

^a^ Statistically significant results (*p*< 0.05) are in bold.

| **Table S2** Correlation analysis of allergic diseases | | | |
| --- | --- | --- | --- |
|  | **Asthma** | **Allergic Rihnitis** | **Eczema** |
| **Asthma** | 1.000 | **.287** | **.154** |
| *p*-value |  | **<.001** | **<.001** |
| **Allergic Rihnitis** |  | 1.000 | **.156** |
| *p*-value |  |  | **<.001** |
| **Eczema** |  |  | 1.000 |

^a^ Statistically significant results (p< 0.05) are in bold.
